# Elevated Serum IgG4 Levels in a Young Patient with Polyserositis and* Necator americanus* Infection

**DOI:** 10.1155/2018/2974756

**Published:** 2018-01-31

**Authors:** Giuseppe D. Sanna, Roberto Manetti, Valentina de Filippo, Sergio Babudieri

**Affiliations:** ^1^U.O.C. Cardiologia, Ospedale “SS Annunziata” AOU Sassari, Via Enrico De Nicola, 07100 Sassari, Italy; ^2^U.O.C. Clinica Medica AOU Sassari, Viale San Pietro 8, 07100 Sassari, Italy; ^3^Istituto di Malattie Infettive AOU Sassari, Viale San Pietro, 07100 Sassari, Italy

## Abstract

IgG4-related disease is a fibroinflammatory systemic condition characterized by tumefactive lesions, lymphoplasmacytic infiltrate rich in IgG4-positive plasma cells, storiform fibrosis, and elevated serum IgG4 concentrations. It has been described in virtually every organ system. Autoimmunity and infectious agents are potential immunologic triggers in IgG4-related disease. Herein, we describe a peculiar case of effusive-constrictive pericarditis in an 18-year-old boy with polyserositis and concomitant* Necator americanus* infection.

## 1. Introduction

IgG4-related disease is a systemic fibroinflammatory disease with tissue infiltration of IgG4-positive plasma cells. The histopathological features show similarities across organs; however clinical features may vary considerably. Cardiac manifestations may include pericarditis, often in a constrictive form.

## 2. Case Presentation

An 18-year-old boy immigrant from Guinea was admitted to our hospital because of fever and abdominal pain starting about three weeks before. His past medical history was unremarkable. Physical examination revealed diminished lung breath sounds consistent with pleural effusion and hepatomegaly. Lab tests showed abnormal blood count (PLT 741 × 10^3^/mm^3^), high erythrocyte sedimentation rate (ESR—87 mm/h), C-reactive protein (CRP—10.22 mg/dl), and gamma globulins (25.3%, n.v. 11.1–18.8%), while procalcitonin (PCT) was 0.9 ng/ml (n.v. < 0.5 ng/ml). All these findings suggested an inflammatory/infectious disease.

**Figure 1:**
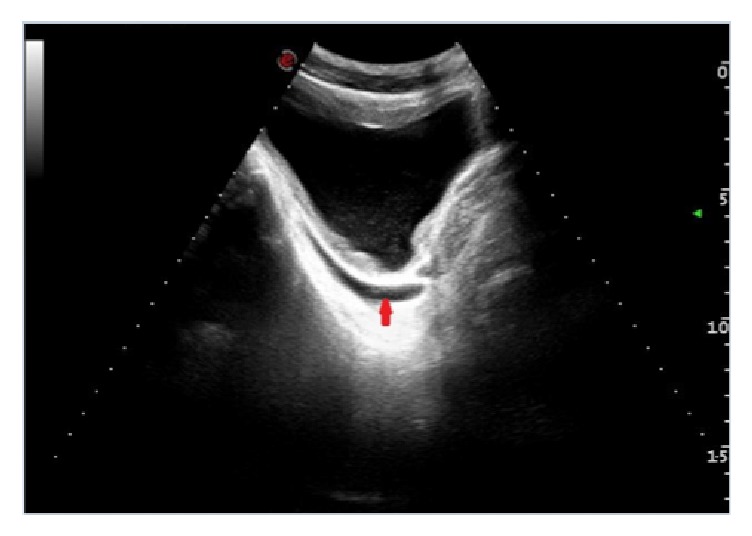
*Abdominal ultrasound.* Bladder wall thickening (red arrow).

**Figure 2:**
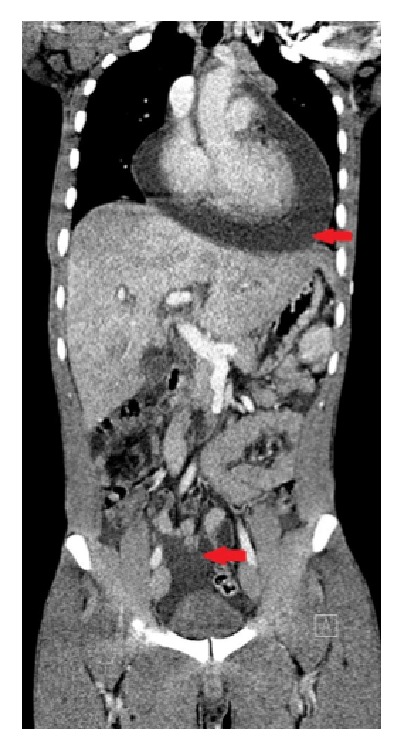
*Computed tomography (CT scan).* Fluid accumulation within the pericardium and abdomen (red arrows).

Ultrasound examination showed fluid in the abdominal cavity together with bladder wall thickening (Figure 1), hepatomegaly, and pleural and pericardial effusion. Computed tomography confirmed all these findings (Figure 2).

**Figure 3:**
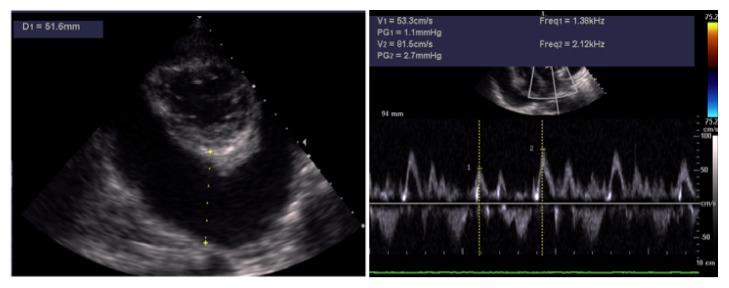
*Transthoracic echocardiography*. Parasternal short axis view (apex) showing a large pericardial effusion (>5 cm) with a swinging heart. Pulse wave Doppler (PWD) examination shows significant respiratory variations (i.e., >25%) in left ventricular diastolic filling pattern.

**Figure 4:**
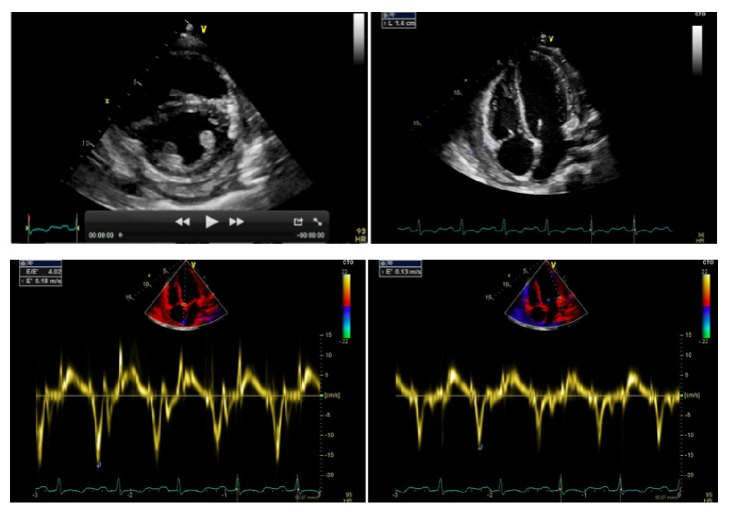
*Transthoracic echocardiography.* Constrictive pericarditis. Pericardial thickening with fibrin deposits (14 mm), ventricular interdependence (“septal bounce”), and abnormal tissue Doppler imaging (“annulus paradoxus” and “annulus reversus”).

**Figure 5:**
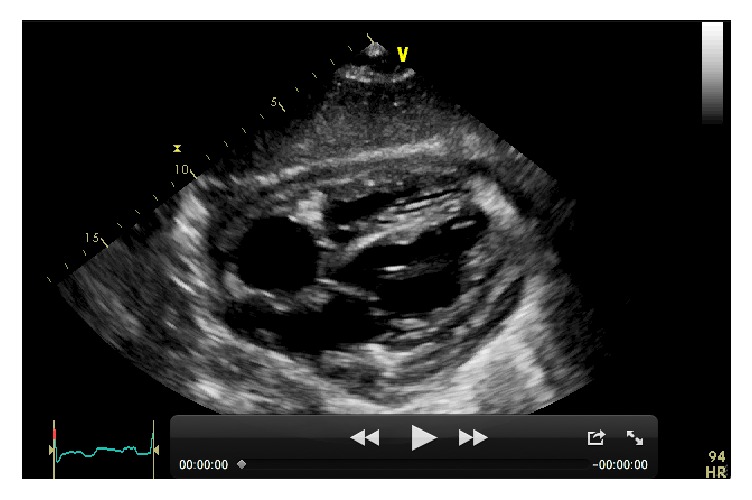
*Transthoracic echocardiography.* Constrictive pericarditis. Pericardial thickening with a large amount of fibrin totally encasing the heart.

Within few days the patient experienced worsening fatigue and dyspnea. Significant jugular venous distension and hepatomegaly were present on physical examination. Blood pressure was 100/60 mm Hg without evidence of pulsus paradoxus. However, echocardiography showed a large pericardial effusion (>5 cm) with significant respiratory variations (i.e., >25%) of mitral inflow and inferior vena cava plethora (Figure 3). All these findings were considered consistent with an incipient cardiac tamponade and the patient was treated with an urgent echo-guided subxiphoid pericardiocentesis (1500 mL of serohematic fluid), without complications. A pericardial drainage catheter was left in situ up to 48 h in order to promote adherence of pericardial layers and prevent further accumulation of fluid. The analysis of pericardial fluid was unremarkable (e.g., serology negative for* Mycobacterium tuberculosis*). The patient was empirically treated with Ibuprofen (600 mg tid) and Doxycycline (100 mg bid), until further microbiological tests (faeces and urine) revealed an* Ancylostoma duodenale*/*Necator americanus* infection. The patient was thus successfully treated with mebendazole (100 mg bid). His clinical conditions progressively improved, despite the persistence of abdominal discomfort. A new echocardiogram after 6 weeks showed an evolution to constrictive pericarditis (Figures 4 and 5).

**Figure 6:**
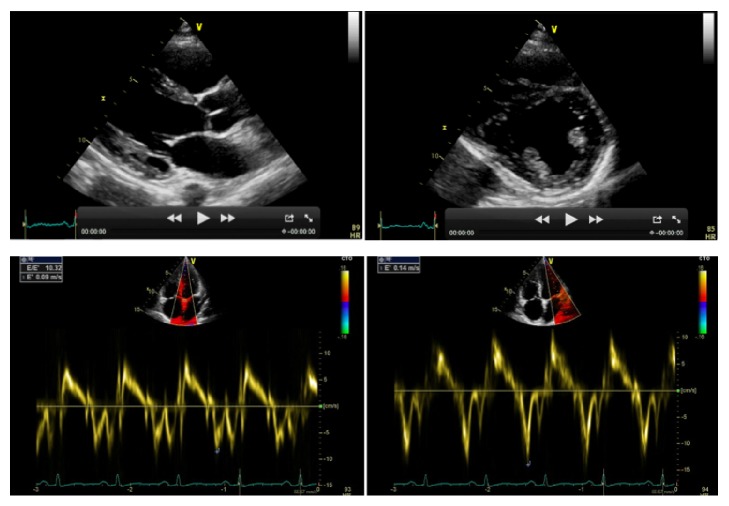
*Transthoracic echocardiography.* Almost complete resolution of the pericardial disease after 5 weeks of therapy with glucocorticoids.

At this point, due to the lack of a definite aetiology for the polyserositis with effusive-constrictive pericarditis we suspected an IgG4-related disease. Our diagnostic hypothesis was confirmed by elevated serum IgG4 concentrations (3.050 g/L, n.v. 0.011–1040). The patient was thus treated with prednisolone at a dose of 75 mg per day for 2 weeks. The echocardiogram showed a progressive resolution of the pericardial disorder without constrictive physiology. The prednisolone was tapered to 25 mg per day and the patient was discharged. Last follow-up echocardiogram (three weeks later) showed an almost complete resolution of the pericardial disease with mild residual thickening of both layers and residual deposits of fibrin (Figure 6).

## 3. Discussion

IgG4-related disease is an increasingly recognized systemic immune-mediated condition. Its features include tumor-like swelling of involved organs, a lymphoplasmacytic infiltrate rich in IgG4-positive plasma cells, and a variable degree of fibrosis that has a characteristic “storiform” pattern [1]. Furthermore, approximately 60 to 70 percent of patients with IgG4-related disease have elevated serum concentrations of IgG4 [2].

Since there is still no definitive serological test to diagnose IgG4-related disease, a combination of histological, immunophenotypic, clinical, radiographic, and laboratory investigations has been proposed to perform the diagnosis [3]. For example, the diagnosis of IgG4-related tubulointerstitial nephritis requires the histological feature of tubulointerstitial nephritis rich in plasma cells with increased IgG4-plasma cells and at least one other characteristic of the categories of “imaging,” “serology,” or “other organ involvement” [3].

In a recent large cohort study the histopathological evaluations were more frequently performed in the most easily accessible organ such major salivary glands, kidney, lacrimal glands, and lung; in contrast, only a minority of patients underwent pancreas or retroperitoneal/periaortic biopsy because these deeper structures are much more difficult to access [4].

The same authors showed elevated serum levels of IgG4 in more than 95% of Japanese patients with IgG4-related disease [4]. In contrast, Wallace et al. showed that only 51% of patients had elevated serum IgG4 concentrations. In this study, 76% were Caucasian and only 8.8% were Asian [5]. Therefore, the difference in the proportion of patients with elevated serum IgG4 in these studies may be due at least in part to racial differences. Another important factor possibly influencing the frequency of patients with high serum IgG4 is the proportion of patients with single organ involvement in each study. In the study by Wallace et al. [5] 38% of patients had single organ involvement, in contrast to 11% in the study by Yamada et al. [4].

IgG4-related disease is now a well, although rare, recognized cause of pericardial disease (i.e., constrictive pericarditis) with several reports over the last years [6, 7].

We would like to draw attention to certain key aspects of our report, first of all the clinical course of the pericardial disease in our patient. Pericardial constriction due to IgG4-related disease, once clinically manifested, often requires biopsy followed by surgical pericardiectomy. In our patient we were planning these procedures due to the worsening of pericardial pathology; however, we observed an impressive and prompt response to glucocorticoids with an almost complete resolution of the pericardial constriction after 5 weeks, as documented by the instrumental echocardiographic follow-up.

The second aspect is related to the pathophysiology of IgG4-related disease in our patient. The pathogenesis of IgG4-related disease is poorly understood. Autoimmunity and infectious agents are potential immunologic triggers in IgG-4-related disease [1]. Some studies have suggested a possible role for molecular mimicry involving* Helicobacter pylori* [8]. More recently elevated serum IgG4 levels were reported in two cases of pulmonary* Paragonimus westermani* infection [9]. Elevated IgG4 levels were described in helminth infections. All these reports may suggest that parasitic infections are associated with increased serum IgG4 levels but also an immune response triggered by the infection (e.g.,* Necator Americanus* in our patient) causing the IgG4-related disease.

We conclude by recalling that IgG4-related disease, even if rare, should be considered in the diagnostic workout of patients with polyserositis and effusive-constrictive pericarditis and that there are strict relationships between parasitic infections and elevated serum IgG4 levels. To the best of our knowledge this is the first reported case of constrictive pericarditis in a patient with concomitant elevated serum IgG4 levels and* Necator americanus* infection.
